# Commensal and Pathogenic Bacterial-Derived Extracellular Vesicles in Host-Bacterial and Interbacterial Dialogues: Two Sides of the Same Coin

**DOI:** 10.1155/2022/8092170

**Published:** 2022-02-17

**Authors:** Samira Tarashi, Mohammad Saber Zamani, Mir Davood Omrani, Abolfazl Fateh, Arfa Moshiri, Ahmad Saedisomeolia, Seyed Davar Siadat, Stan Kubow

**Affiliations:** ^1^Microbiology Research Center, Pasteur Institute of Iran, Tehran, Iran; ^2^Mycobacteriology & Pulmonary Research Department, Pasteur Institute of Iran, Tehran, Iran; ^3^Immunoregulation Research Center, Shahed University, Tehran, Iran; ^4^Department of Medical Genetics, Shahid Beheshti University of Medical Sciences, Tehran, Iran; ^5^Laboratory of Experimental Therapies in Oncology, IRCCS Istituto Giannina Gaslini, Genova, Italy; ^6^Department of Cellular and Molecular Nutrition, School of Nutritional Sciences and Dietetics, Tehran University of Medical Sciences, Tehran, Iran; ^7^School of Human Nutrition, McGill University, 21, 111 Lakeshore, Ste. Anne de Bellevue, QC, Canada H9X 3V9

## Abstract

Extracellular vesicles (EVs) cause effective changes in various domains of life. These bioactive structures are essential to the bidirectional organ communication. Recently, increasing research attention has been paid to EVs derived from commensal and pathogenic bacteria in their potential role to affect human disease risk for cancers and a variety of metabolic, gastrointestinal, psychiatric, and mental disorders. The present review presents an overview of both the protective and harmful roles of commensal and pathogenic bacteria-derived EVs in host-bacterial and interbacterial interactions. Bacterial EVs could impact upon human health by regulating microbiota–host crosstalk intestinal homeostasis, even in distal organs. The importance of vesicles derived from bacteria has been also evaluated regarding epigenetic modifications and applications. Generally, the evaluation of bacterial EVs is important towards finding efficient strategies for the prevention and treatment of various human diseases and maintaining metabolic homeostasis.

## 1. Introduction

Extracellular vesicles (EVs) are nanosized membrane-encapsulated cell fragments shed from different domains of life, including bacteria [1]. The first evidence of bacteria-derived EVs dates back to the 1960s [2–4]. However, researchers initially failed to realize the importance of these newly discovered structures, which were mistakenly identified as unused cellular components ejected as “trash cans” [1, 5]. Another misconception was the assumption that bacteria-derived EVs constitute cellular debris solely from decomposition of dead cells, whereas in reality, the production of EVs by bacteria requires their metabolic activity [6, 7]. Different routes of formation of bacterial EVs have been indicated based on the diverse cell wall architecture of the EVs, which includes outer membrane vesicles (OMVs), inner membrane vesicles (IMVs), outer-inner membrane vesicles (O-IMVs), explosive outer membrane vesicles (EOMVs), cytoplasmic membrane vesicles (CMVs), and tube-shaped membranous structures (TSMSs). These latter EVs contrast to bacterial EVs formed during phage endolysin-triggered cell lysis as bacteria-derived EVs originating from membrane blebbing of living cells are structurally different from EVs derived from cell lysis [8–12]. This latter distinction in the structure of EVs is associated with differences in their components, and possibly, their functions [12]. In that regard, such released membranous structures cannot replicate but are involved in the shuttling of bioactive compounds from their mother cells, including proteins, lipids, nucleic acids, and metabolites [13].

Trillions of different bacterial EVs are produced by not only pathogenic bacteria during infections but also by commensal microbiota communities that are colonized in various niches of a normal human body, particularly mucosa [14]. These nanosized bacterial vesicles act as major mediators in host-bacterial interactions by transporting different molecules, which contribute to physiological and pathophysiological processes [15–17]. Remarkably, bacteria-derived EVs are involved in interbacterial interactions [18, 19]. Additionally, these vesicles may be transferred to different organs of the host via systemic circulation to promote communication under homeostatic or pathogenic conditions [20]. Although the definitive role of bacterial EVs in affecting homeostatic and pathological conditions of the host is not yet fully understood, there is increasingly evidence supporting this connection [1].

Bacterial EVs are extensively studied *in vitro* using cell line cultures isolated from a mixture of vesicles released by different types of bacteria or from a specific microorganism. Since bacterial EVs are similar to eukaryote-derived EVs in size, it is difficult to differentiate them based on size alone [21]. Besides, the size and composition of bacterial EVs can vary drastically, depending on the growth conditions and type of strain (even within the same species) [22–24]. Moreover, knowledge of specific markers present specifically on bacterial EVs is limited. Generally, lack of a standardized methodology for purification and isolation of bacterial EVs is one of the major limitations, deterring progress in this field [21]. This lack of knowledge of specific EV markers and EV cargos, as well as EV biogenesis, is an important challenge for clinical researchers [6, 25]. Also, the evaluation of the understanding of different possible functions of bacterial EVs presents even a greater challenge. Despite limited knowledge about these bioactive molecules as compared to eukaryotic EVs, studies on bacterial EVs are continuously increasing as the possible protective or adverse effects of bacterial EVs in interbacterial and host-bacterial interactions has engendered great research interest. Therefore, this review article is aimed at addressing the current status of research on bacterial EVs by focusing on their role in host-bacterial interactions that affect host homeostasis and pathogenesis. Overall, the knowledge of the pertinent mechanisms for the above relationships may lead to the development of new therapeutics and diagnostics using bacterial EVs.

## 2. Subcategories of Bacterial Derivative Vesicles

Bacterial EVs are typically stable vesicles, which can be classified into several subcategories based on differences in the bacterial cell wall structure, Gram-positive or Gram-negative bacteria. The characteristics of different types of bacterial EVs are shown in Table 1. A peptidoglycan-rich cell wall is found in Gram-positive bacteria, while in Gram-negative bacteria, the outer and inner membranes contain lipopolysaccharides (LPS). Therefore, the biogenesis of vesicles derived from Gram-positive and Gram-negative bacteria is probably different. Although no single mechanism of bacterial EV release has been identified, one model suggests that Gram-negative bacteria randomly release EVs as side products of normal cellular processes related to the cell wall turnover. During this recycling process, the outer membrane budding and subsequent OMV formation can result from the loss of interaction between the outer membrane and peptidoglycans. Various routes can weaken this latter interaction to form OMVs. For example, imbalanced turnover of peptidoglycans, accumulation of phospholipids, and diacylation of lipid-A in LPS can induce OMV blebbing. The other mechanisms include intercalation of hydrophobic substrates such as antibiotics and bacterial signaling molecules into the outer membrane and the shedding of membrane blebs from flagella. Shedding of membrane blebs from the flagella is a unique mechanism based on the alteration of membrane-sheathed flagella by which OMVs are released when the flagella are rotating [26]. Structurally, OMVs derived from Gram-negative bacteria contain LPS [17, 27]. Moreover, a structural change in LPS may lead to membrane deformation and bacterial EV shedding [28]. Therefore, it is recognized as a major route of LPS release and inflammation induction can occur without bacterial killing [29]. It is also important to consider that the release of LPS does not constantly trigger inflammatory responses as this feature is dependent on the LPS type, including proinflammatory LPS (P-LPS) or anti-inflammatory LPS (A-LPS). P-LPS is often present in pathogenic bacteria, inducing strong proinflammatory responses, septic shock, and even death, as traditionally described. On the other hand, A-LPS is mainly formed by certain commensal microbiota, inducing antagonistic activity to inhibit proinflammatory responses [30]. The biological activity of P-LPS is associated with differential immune activation by various bacterial species, while the basic structure and chemical properties are generally similar. Interestingly, the functional difference between various LPS is affected by structural variations of O-antigen and lipid A (number of phosphate groups, number, and length of acryl chains) [31].

In a stricter clarification of bacterial EVs, some Gram-negative bacteria release a different type of EV, called inner membrane vesicles (IMVs). IMVs are formed by fissioning the protrusion of the outer and plasma membranes and entrapping the cytoplasm components. The outer IMVs (O-IMVs) are also formed as double-layered EVs, originating from cytoplasmic turgor pressure and containing most DNA fragments of Gram-negative bacteria. O-IMVs are also frequent products of weakening of the peptidoglycan layer by hydrolysis and protruding of the inner membrane into the periplasm. In this manner, cytoplasmic content such as DNA fragments are packed into O-IMVs. In addition, formation of Gram-negative bacterial EVs that are highly contained by cytoplasmic content and DNA fragments could be mediated through explosive cell lysis. Once cell lysis is triggered and peptidoglycan is degraded, the cell explodes and the devastated cell envelope fragments round up and reassemble into “explosive outer membrane vesicles” (EOMVs) that enclose the released DNA fragments [12]. In addition, tube-shaped membranous structures (TSMSs) have been recently introduced as a particular type of bacterial EV, protruding from the cell surface of Gram-negative or Gram-positive bacteria. TSMSs are often involved in the formation of intercellular connections between neighboring cells to facilitate the exchange of various cellular components. The difference between these tube-like structures in Gram-negative and Gram-positive bacteria depends on their origin membrane and, consequently, transfer of different components. In Gram-negative bacteria, TSMSs originate from the outer membrane, which enables the intercellular transfer of membrane proteins, periplasmic metabolites, and lipids, but not cytoplasmic content. On the other hand, in Ggram-positive bacteria, the TSMSs are derived from the cytoplasmic membrane and exchange various cytoplasmic content, such as proteins and plasmid DNAs. Cytoplasmic microvesicles (CMVs) are another specific type of Gram-positive bacterial EVs. It was previously assumed that EVs cannot be released through the thick cell wall of Gram-positive bacteria. However, the formation of EVs has been indicated to occur via pressure through pores in the cell wall, a conservative blebbing mechanism from the cell membrane, or by degradation of the cell wall of Gram-positive bacteria [12, 32]. Some proteins, such as peptidoglycan-degrading enzymes, phenol-soluble modulins, and autolysins, increase the cell membrane fluidity and facilitate CMV release [9, 33, 34]. Generally, the formation of different EVs by both Gram-positive and Gram-negative bacteria is based on two principal processes, namely, shedding from living cells and endolysin-triggered cell lysis [12]. The shedding of bacterial EVs from living cells is an active metabolic process in constantly living cells, while endolysin-triggered cell lysis is based on the enzymatic activity that enables the lysis of original cells. During this process, double-stranded DNA phages lyse their host cells until phage progeny can be released; consequently, the shattered membrane fragments round up and self-assemble into EVs [12].

The profiles of bacterial EVs and the original bacterial membrane fractions do not necessarily match, as some specific cargos may be actively sorted into bacterial EVs [35]. In models of active biogenesis of bacterial EVs, the vesiculation of EVs mainly occurs in distinct areas of the cell membrane, known as “hot spots.” These specific regions are locally enriched with specific lipids and proteins involved in hypervesiculation, while vesiculation inhibitory proteins, such as lipoproteins needed for cell wall integrity, are reduced [1, 36]. Also, some proteins affect vesiculation by deleting genes involved in EV formation. Nevertheless, the active biogenesis of bacterial EVs is not still fully understood, and different bacterial strains possibly use different vesiculation mechanisms [37, 38]. The fission of formed vesicles, as the final vesiculation step, is an active process that requires energy, while there is no energy source in the periplasm; therefore, it seems that conformational changes of the outer membrane proteins are involved [36]. The continuous folding of outer membrane proteins and their conformational changes provide the required energy for vesiculation.

The biogenesis of bacterial EVs can vary under specific conditions, which can affect their properties. Changes in temperature, nutrients, and stress exposure are some of these above conditions [1]. EVs are released from pathogens that encounter numerous stressors during colonization [39]. Antibiotic agents may even stimulate EV shedding through various mechanisms, depending on the antibiotic [12]. Also, several environmental stressors, such as nutrient deficiency, oxidative stress, UV radiation, pH changes, heat shock stress, osmotic pressure, hydration, and desiccation, as well as the host immune system, increase EV shedding [39]. Under these latter conditions, peptidoglycans or misfolded proteins accumulate excessively in the periplasm through the effects of physical or chemical stress-induced malfunctioning membranes. This phenomenon increases turgor pressure, membrane protuberances, and pinching-off of small membrane portions [40]. Through this mechanism, bacteria can release additional proteins into the extracellular space to combat stressors and survive [1].

Differences in bacterial EVs go beyond the cell wall structure and may also depend on their compositional differences (related to the bacterial cell origin). The EV components may include lipids and proteins (toxins and enzymes), causing differences in EV functionality [41]. As described earlier, the cargo composition of bacterial EVs is not usually similar to the bacterial origin, and specific cargos can be actively sorted within them [35]. The high plurality of bacterial EVs and the lack of universal or common markers for all bacterial EVs may be related to the diversity of bacterial origins [42]; even in cultures with similar conditions, different bacterial EVs can be identified, which is a puzzling phenomenon [43]. A consequential difference between bacterial EVs, which is particularly important for studying their interactions with various cells, is based on the commensal or pathogenic bacterial origin [6, 21]. The study of bacterial EVs has focused mainly on Gram-negative pathogenic bacteria, while the mechanisms regulating the EV release under homeostatic and pathogenic conditions remains hypothetical. A problem with bacterial EVs is difficulty understanding their primary acute or chronic functional biological properties [17]. In the following sections, the importance of commensal or pathogenic bacterial EVs in host-bacterial and interbacterial interactions to maintain homeostasis or in the development of pathogenesis of the host will be highlighted.

## 3. The Importance of Bacterial EVs in Host-Bacterial Dialogues

**Figure 1:**
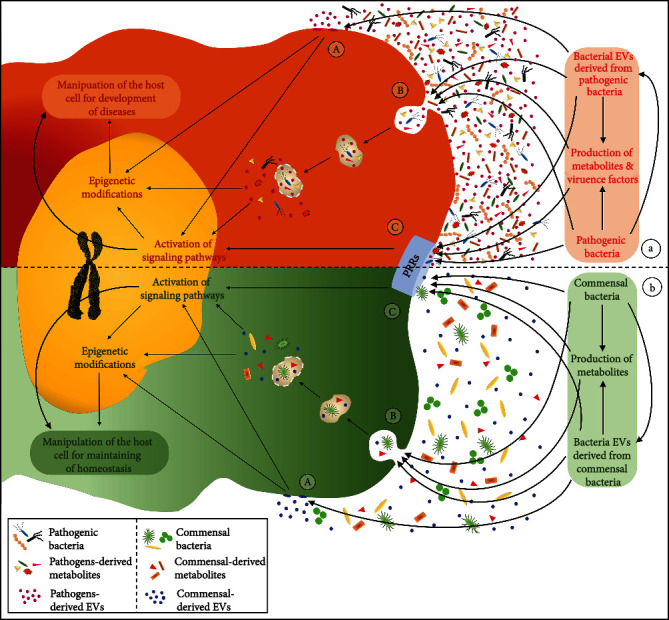
The schematic comparison of pathogenic or commensal bacterial EV importance in host-bacterial interactions to develop homeostasis or pathogenesis conditions. (a, A, b, A) Fusion of pathogenic or commensal bacterial EV with host cell membrane. Direct release of components in the cytoplasm and impact on signaling pathways or epigenetic modifications may develop pathogenesis or homeostasis conditions. (a, B, b, B) Direct entrance of pathogenic or commensal bacteria, their metabolites, or derived EVs to the host cell. Impact of such bacteria, metabolites, or components of bacterial EVs on signaling pathways or epigenetic modifications may also develop pathogenesis or homeostasis conditions. (a, C, b, C) Activation of PRRs by pathogenic or commensal bacteria, their metabolites, or derived EVs. Activation of PRRs to stimulate signaling pathways may directly develop pathogenesis or homeostasis conditions or indirectly develop such conditions by epigenetic modifications. Three main mechanisms of uptake of bacterial EVs by host cells are indicated in Figure 2 in details.

**Figure 2:**
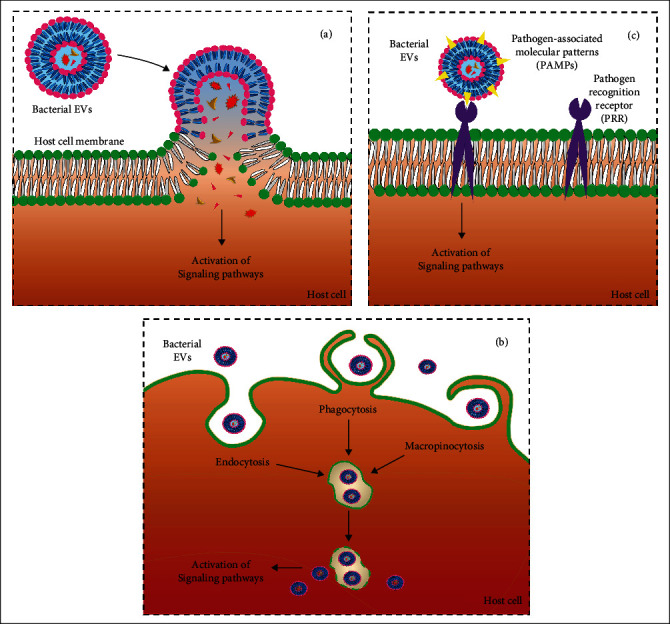
Three main mechanisms of EV uptake by host cells in schematic. (a) Delivery of bacterial EV contents into the host cell by direct membrane fusion or lipid rafts. (b) Direct entrance of bacterial EVs by endocytosis, phagocytosis, or micropinocytosis. (c) Ligand-receptor interaction. The order of mechanisms is in parallel to Figure 1.

As mentioned earlier, bacterial EVs involve interactions between bacteria and host cells. Since the community of commensal or pathogenic bacteria and their metabolites can directly or indirectly induce positive or negative host health effects, the derived EVs are also critical to maintaining homeostasis or pathogenesis (Figure 1). There are several mechanisms involved in the uptake (Figure 2) and effects of EVs on the immune system. Briefly, the mechanisms of interactions between bacterial EVs and host cells include EV interactions with the host receptors, delivery of EV cargos to the host cell, and full incorporation of EVs into the host cell cytoplasm [6]. Several mechanisms, including endocytosis, phagocytosis, micropinocytosis, internalization through lipid rafts, direct membrane fusion, and ligand-receptor interactions, have been proposed for the uptake of EVs [17, 44]. Moreover, Toll-like receptors (TLRs) and NOD-like receptors (NLRs) are critical pattern recognition receptors (PRRs), involved in direct host-bacterial interactions through bacterial EVs [45]. The involvement of TLR2 in the internalization process of EVs derived from *Moraxella catarrhalis* and *Mycobacterium* in epithelial cells has been established [46, 47]. The interaction of *Bifidobacterium* and *Lactobacillus*-derived EVs with dendritic cells enhances TLR2/1 and TLR4 responses [48]. Similarly, cellular LPS-binding proteins (LBPs) are important in picking up bacterial EVs exposing LPS [49].

Abundant evidence has confirmed the effects of pathogen-derived EVs in host-bacterial interactions to facilitate the interaction of pathogens with their hosts by acting as intermediates, inducing pathogenic protection and host immunomodulation as extracellular virulence factors [50–53]. Bacterial EVs can also act as an effective secretory and delivery system to transfer various molecules to bacteria and host cells, regardless of the physicochemical structure [53]. These vesicles also function as a delivery system to transfer various virulence factors, including degradative enzymes and toxins, possibly leading to immunosuppression [54]. The released EVs from *Staphylococcus aureus* stimulate proinflammatory cytokines and facilitate immune responses [50]. *Clostridium perfringens-*derived EVs increase interleukin- (IL-) 6, tumor necrosis factor- (TNF-) *α*, and granulocyte colony-stimulating factor (G-CSF) expression to stimulate inflammation [55]. The released EVs during infection with mycobacterial species stimulate TLR2, TLR4, and Myd88-dependent signaling pathways to induce IL-12 and TNF-*α* production involved in proinflammatory responses [56].

In contrast, EVs derived from commensal microbiota have benefits for host-bacterial interactions, such as inhibition of pathogenic colonization and regulation of immune system responses. Under hemostatic conditions, bacterial EVs derived from the commensal microbiota community are prominent, leading to the suppression of pathogenic colonization [21]. Mounting evidence suggests the immunomodulatory and anti-inflammatory roles of commensal EVs [57, 58]. EVs released from *Akkermansia muciniphila* inhibit the production of IL-6 during colitis [59]. Also, *Bacteroides fragilis*-derived EVs, carrying polysaccharide A, are sensed by TLR2 in intestinal immune cells. Besides, *Bacteroides thetaiotaomicron*-derived EVs containing hydrolytic enzymes improve digestion by sharing these components [60].

Despite the beneficial effects of these commensal EVs, they may also disrupt host-bacterial interactions, as they can transfer virulence factors, such as antibiotic resistance genes, to pathogenic bacteria [61]. For instance, *Bacteroides thetaiotaomicron* and several other *Bacteroides* species, as a major part of the gut microbiota, encode *β*-lactamase. On the other hand, the produced bacterial EVs contain *β*-lactamases that can enhance cefotaxime resistance in both commensal (such as *B. breve*) and pathogenic bacteria (such as *Salmonella typhimurium*). Therefore, the production of bacterial EVs by commensal microbiota may positively affect not only commensal microbiota but also pathogenic bacteria [61]. Overall, shedding of bacterial EVs depends on the bacteria or host cell type producing heterogenic EVs regarding size, content, and composition of EV cargos, which may ultimately affect the host immune response [5]. Various exogenous and endogenous factors can impair the composition of commensal microbiota, such as dysbiosis induction and increased colonization of pathogens and so the production of bacterial EVs may change accordingly. Abnormal bacterial EVs may facilitate host pathologies via interactions with host cells or by affecting the normal microbiota composition. There is growing evidence that bacterial EVs from dysbiotic microbiota can cross the altered epithelial barrier and enter systemic or lymphatic circulation to gain access to distant tissues and interact with immune cells in the body [62]. In this manner, bacterial EVs may interact with various axes in the body.

## 4. The Importance of Bacterial EVs in Various Axes

Mucosal and epithelial barriers in the tissues and organs represent a significant defense line to protect against harmful external stimuli. These host barriers are derived from epithelial and endothelial cells, organized by various tight junction proteins, along with other supporting structures that maintain their integrity [63]. Disruptions in these host barrier structures have been implicated in various disorders. While several factors influence the host barrier, recently, there has been a growing interest in the role of gut bacteria-derived EVs in regulating the barrier integrity, which plays an important role in various axes of the body. In that regard, bacterial EVs have been observed in human plasma and cerebrospinal fluid [62]. Several studies have confirmed the importance of bacterial EVs in the gut-brain, gut-liver, and gut-lung axes [64–66]. Therefore, the effects of bacterial EVs on epithelial differentiation and integrity in various axes of the human body are of particular interest. It has been reported that EVs released from *Vibrio cholerae* stimulate gene expression in association with epithelial cell differentiation [67]. Likewise, isolated EVs from some commensal *Escherichia coli* strains, such as EcN and ECOR63, upregulate the tight junction proteins claudin-14 and zonula occludens- (ZO-) 1 and downregulate claudin-2 (a leaky protein) [68]. The epithelial barrier is responsible for suppressing leaky gut and endotoxemia that lead to inflammation. As described earlier, in addition to the effects of bacterial EVs on epithelial differentiation and integrity, their ability to transfer to different organs via systemic circulation has attracted the research attention [20]. Different axes, especially the gut-brain and gut-liver axes, have been proposed to promote communication in the human body under homeostatic or pathogenic conditions by transferring bacterial EVs from their origin to other organs.

Three probable mechanisms are indicated to be used by bacterial EVs to penetrate into the target and distant organs [69]. The transfer of bacterial EVs from their origin to the target organ, by directly crossing epithelial and endothelial barriers, is one of the possible mechanisms. The crosstransfer of bacterial EVs through the blood-brain barrier has been also confirmed. Different bacteria may translocate to various organs via circulation and release their EVs in the target organs; this process is the second transfer mechanism. The final possible mechanism involves the infection of immune cells by bacterial EVs in the origin organ and transfer by infected cells to the target organs. This mechanism may be similar to the defined “Trojan horse” transfer of bacteria to escape unfavorable conditions and immune system responses [70]. Some studies have highlighted the importance of bacterial EVs in different organs. A previous study confirmed the biological effect of *A. muciniphila*-derived EVs on the enhancement of serotonin production in the colon and hippocampus of mouse models and Caco-2 cell lines [64]. A similar study established that treatment of *Faecalibacterium prausnitzii* and *A. muciniphila*-derived EVs affected the expression of genes involved in serotonin release in the Caco-2 cell line [71]. Moreover, EVs from *Bacillus subtilis* were shown to be transported across Caco-2 cells to be secreted at the opposite cell surface side [72]. Besides, the positive effects of *A. muciniphila*-derived EVs on the attenuation of inflammatory cytokine expression and subsequent prevention of liver fibrosis has been described [65]. Moreover, the efficiency of EVs derived from *A. muciniphila* has been established in restoring the barrier integrity, lipid metabolism, modulation of obesity, and promotion of homeostasis [73]. The improvement of various disorders, such as metabolic diseases, cancers, gastrointestinal, mental, and psychiatric disorders, by prescription of bacterial EVs and microbiota manipulation via diet has been postulated [69, 74, 75]. Regarding the important effects of bacterial EVs on different bidirectional axes, further studies are needed to unravel novel therapeutic processes in various disorders to restore homeostasis.

Considering the putative effective roles of bacterial EVs, any changes in their production can affect their host interactions. It has been confirmed that pathogenic bacteria release significantly more EVs compared to commensal microbiota [76]. Undeniably, the infection type (acute or chronic) is important in the efficiency of bacterial EVs. Acute infectious pathogens rapidly colonize, proliferate, and spread in the host, whereas chronic infectious pathogens proliferate and spread less rapidly, cause long-term infections, and may persist for longer periods [77]. This phenomenon is of great significance in chronic infections, where persistent pathogens can release significantly more EVs during infection, thereby manipulating the host cells for immune evasion, survival, and persistence [78].

## 5. The Importance of Bacterial EVs in Epigenetic Modifications

The evaluation of the importance of bacterial EVs in host-bacterial interactions is multifaceted, and there are still many vague aspects. Recently, an interkingdom crosstalk was established between host and bacterial cells through epigenetic modifications by bacterial EVs (Figure 1). It is obvious that the induced epigenetic modifications by bacterial EVs and their components are more intricate than they first appeared. Interestingly, such vesicles may package noncoding RNAs, as well as other components, which can function as epigenetic regulators in the recipient host cells [79]. Noncoding RNAs, such as microRNAs, are among these epigenetic modifications. Evidence suggests that some bacteria, such as *Streptococcus mutans* and *E. coli*, produce vesicles containing microRNA-like molecules that may impair the “eukaryotic miRNA machinery” to their favor [80, 81]. Also, further evaluation of *E. coli* and *V. cholerae* revealed that RNA is a component of many bacterial EVs and highlighted the potential role of RNA-containing bacterial EVs in the host-bacterial interactions [82]. It is well-established that various bacterial EV components are aligned with histone proteins (such as H3K4Me1, H3K4Me3, and H3K27Ac), chromatin-modifying enzymes, transcription factors, or constitute ribonucleoprotein complexes in host cells for epigenetic regulation [79].

The homeostatic and pathological conditions caused by epigenetic interactions of commensal microbiota or pathogenic bacterial EVs with eukaryotic genomes are not fully understood. Bacterial EVs preserve these fragile cargos against degradation by enzymes and are responsible for selecting host cells [1]. Indirect epigenetic modifications and chromatin accessibility of promoters and transcription start sites (TSSs) of genes (by stimulating bacterial EVs) are associated with commensal microbiota *E. coli* and pathogenic *V. cholerae* in coculturing with colorectal carcinoma cells [67]. Global 5-methylcytosine (5mC) hypermethylation has been also observed in the salivary samples of periodontitis patients, induced by significantly increased levels of EVs from periodontal pathogens (*Porphyromonas gingivalis*, *Fusobacterium nucleatum*, *Eikenella corrodens*, and *Treponema denticola*) [83]. Likewise, salivary bacterial EVs seem to be associated with DNA methylation in IL-8, IL-6, IL-10, IL-1*β*, and TNF-*α* gene promoters in gingivitis patients [84]. Moreover, the transfer and delivery of methyltransferases (DNMT1, DNMT3A, and DNMT3B) by eukaryotic EVs and gene expression regulation through alteration of DNA methylation have been confirmed in the literature [85]. Therefore, bacterial EVs may carry various cell fragments from their origin as epigenome regulators. This host-bacterial interaction supports long-distance bacteria-derived vehicles that may potentially control the host cell response, depending on the tendency of operating bacteria, commensal microbiota, or pathogenic bacteria. These bacterial vesicles may affect epigenetic modifications not only in the host-bacterial interactions but also in interbacterial dialogues that warrant more attention.

## 6. The Importance of Bacterial EVs in Interbacterial Dialogues

Regarding the host-bacterial interactions, bacterial EVs are involved in the interactions of bacterial communities. A complex interaction has been established between commensal or pathogenic bacterial communities and the released bacterial EVs, as an important contributor to different bacterial interactions or competition strategies [86]. The specific bacterial EV cargo components may be also responsible for a particular EV function in the bacterial community, such as antibiotic resistance, biofilm formation, quorum sensing, and virulence factors [17, 87, 88]. These functions of EVs have been reported in both commensal microbiota and pathogenic communities to meet specific goals and increase their survival [20]. Bacterial EVs mediate antibiotic resistance by facilitating horizontal gene transfer, trapping antimicrobial agents, and carrying related enzymes to suppress antibiotic activities. For instance, some carbapenem-resistant strains of *Acinetobacter baumannii* may release EVs containing OXA-24 carbapenemase gene to horizontally transfer carbapenem resistance to other susceptible strains [89]. This horizontal gene transfer is not only a delivery system in bacterial communities but also a way to protect DNA against degradation under environmental stress [90]. The induction of antibiotic resistance may be also induced by trapping of antimicrobial agents in the EV compartments to survive longer in the microbial community. Some studies have confirmed the longer survival of bacteria producing EVs compared to their wild-type counterparts under antibiotic exposure [91, 92]. *S. aureus* and *Haemophilus influenzae*-derived EVs transfer *β*-lactamase to protect *S. epidermis*, *E. coli*, *S. enterica*, and group A streptococci against ampicillin and amoxicillin activities in the bacterial community [25, 93]. Similarly, *Bacteroides* species, as predominant genera in the gut microbiota, produces EVs containing cephalosporinases to induce *β*-lactam resistance [61]. Also, EVs carrying the related enzymes are involved in bacterial protection against possible inactivation [94]. In *M. catarrhalis*, *β*-lactamase packaging in EVs suppresses neutralization by serum IgG [95].

Additionally, numerous components of biofilm matrices (such as alkaline protease, PrpL, and CdrA), quorum-sensing molecules (such as quinolines and lactones), toxins and degradative enzymes, and virulence factors (such as alkaline phosphatase, phospholipase C, lipase, and serine protease) may be carried by bacterial EVs to communicate and coordinate the bacterial community activities [96–98]; carrying such molecules and enzymes can increase the killing of competing bacteria, bacterial invasion, and bacterial adhesion [41, 99, 100]. The extraction of anthrax toxin from the released *Bacillus anthracis-*derived EVs confirms this finding [101]. However, further studies are needed to fully understand the importance of bacterial EVs in interbacterial dialogues.

## 7. Bacterial EV Applications

Although many functions of bacterial EVs have not been elucidated, various properties of bacterial EVs in host-bacterial and interbacterial dialogues modulate defensive or pathogenic functions of EVs. Moreover, bacterial vesicles can be applied to maintain and improve homeostatic conditions. Generally, bacterial EVs are a different type of nonclassical secretory systems with more advanced functions than only the transfer of some cargos to the target sites [1]. In other words, treatment with commensal microbiota-derived EVs for different disorders may further induce the beneficial effects of normal microbiota, as confirmed in leaky gut syndrome [102]. Several beneficial members of commensal microbiota have been introduced into the market as “probiotics,” because of their effects on refining homeostasis [103]. It has been proposed that the derived EVs can mediate the effectiveness of probiotic bacteria, while decreasing the safety concerns and potential risks of consuming living bacteria, as they are nonreplicating [104]. Therefore, such potential activity can also appear in bacterial EVs and introduced them as “postbiotics.” The ability of bacterial EVs to pass through epithelial, endothelial, and blood-brain barriers has highlighted the potential advantages of these delivery molecules in targeted therapy of various infections, diseases, and disorders, especially the gut-brain axis disorders, such as psychopathic disorders [69]. In this regard, application of bacterial EVs has been suggested for the treatment of some disorders such as ulcers caused by *Helicobacter pylori* and inflammatory bowel disease (IBD) [104]. The fundamental functions of these EVs may be associated with their properties as direct and targeted antigen delivery vectors [73]. Therefore, researchers have focused on bacterial EVs as a new biotechnology tool, particularly in cancer treatment.

As mentioned earlier, bacterial EVs mimic their origin cell structure and also contain different immunostimulatory molecules, which have been identified and taken up by immune cells to stimulate immune responses that are beneficial for tumor treatment. In that regard, these nanosized bacterial structures can accumulate in tumors to stimulate and gain local immunity through enhanced permeation and retention effects [105]. The potential of manipulated bacterial EVs is to selectively target tumor cells that may be a novel and specific EV-based therapy [106]. Also, targeting human epidermal growth factor receptor 2 (HER2), which is frequently present in tumor cells, is one of the most important approaches to reduce the tumor burden by manipulating *E. coli*-derived EVs to transfer antitumor components [107]. Bacterial EVs are also directly involved in cancer therapies by altering the microenvironment surrounding the tumor cells. These derived vesicles are specifically involved in extracellular signaling. The administration of bacterial EVs derived from *S. aureus*, *S. enterica*, and *L. acidophilus* can stimulate the expression of tumor-suppressor genes and activate anti-tumor immune responses in tumor tissues [108]. Likewise, bacterial EVs have been introduced as smart vehicles for targeted drug delivery in fundamental biological research. Bacterial EVs are simple targeted delivery systems, which can be coated with targeting ligands by genetically engineering the origin bacteria. Therefore, drug accumulation is facilitated at the target site.

Additionally, bacterial EVs can passively accumulate in tumor sites through enhanced permeation and retention effects because of their size, which is essential for drug delivery to the tumor site. Also, bacterial EVs act as drug delivery vehicles, where the loaded drug protects against denaturation and degradation until reaching the target site. Finally, bacterial EVs as drug delivery vehicles may be known as “foreign” agents, eliciting inflammatory responses and causing diverse effects in the body. Accordingly, detoxified bacterial EVs are suggested to diminish reactogenicity, inflammatory responses, and self-damage; the safety of these bacterial EVs has been confirmed in a mouse model [109, 110].

On the other hand, bacterial EVs can be easily identified and taken up by neutrophils. In other words, circulating neutrophils can be used as cellular carriers to transport bacterial EVs for targeted drug delivery [111]. Overall, advances in the use of bacterial EVs in drug delivery systems have increased their potential clinical applications. Regarding the characteristics of bacterial EVs, with delivery of a sublethal dose of antibiotics, *A. baumannii* infection was successfully treated with the fewest indiscriminate side effects in the commensal microbiota in a mouse model [112]. Also, these effective bacterial vectors can be applied in the direct codelivery of antigens and adjuvants to host cells. Bacterial EVs are suitable adjuvants that induce immune responses to target antigens through different approaches. Three main examples of these approaches include genetic engineering of bacteria to express the target antigens, loading of target antigens on surfaces or in bacterial EVs, and mixing with the target antigens. Following immunization, bacterial EV adjuvants initiate more robust immune responses in terms of quality and quantity as compared to immunization with only purified proteins and antigens [105]. Generally, adjuvants play a role in increasing antigen presentation and uptake, antigen delivery to lymph nodes, and direct stimulation of immunity [113].

Bacterial EVs have been also introduced as a novel vaccine delivery technology to trigger long-lasting and robust immune responses [114]. A vaccine must at least contain the target antigens and several pathogen-associated molecular patterns (PAMPs) and have an appropriate size comparable to the pathogen. Interestingly, bacterial EVs simultaneously have all of the three properties described above [105, 115]. The released EVs from some bacterial species, present in the commensal microbiota or pathogenic bacterial community, may act as a permanent natural vaccine by triggering both innate and adaptive immune responses [57, 116]. For instance, the EVs isolated from *Neisseria meningitidis*, *A. baumannii*, *S. pneumoniae*, *B. anthracis*, and even *M. tuberculosis* can induce a protective immune response to inhibit the development of infection [21, 116]. To date, the administration of only one bacterial EV-based vaccine (MeNZB vaccine) has been approved for human use [117]. MeNZB vaccine has been effectively and safely implemented to combat meningococcal serotype B disease, caused by *N. meningitides*, to control the disease epidemic in New Zealand [118].

Primarily, bacterial EVs are used as adjuvants to trigger an immune response to meningitis B vaccine. They can also deliver some antigens, especially to the target pathogens, such as PorA [119, 120]. PorA is highly variable between different *N. meningitidis* strains and has been introduced as the main immunogenic protein in EVs. Therefore, immunization with bacterial EV vaccines is strain-specific, and use of single strain-derived EVs can restrict vaccine application in an epidemic triggered by several strains. Depending on the need, bacterial EV-based vaccines containing multivalent PorA have been established using the released EVs from bioengineered *N. meningitidis* strains, including multiple *PorA* genes [121, 122]. Along with porins, other minor proteins in bacterial EVs also induce pathogen-specific immunization [123]. Apart from the bacterial EV vaccination approved for *N. meningitidis*, similar vaccines against other pathogens such as *H. pylori*, *S. typhimurium*, *V. cholera*, and *Shigella flexneri* have been evaluated in animal models, as well; however, none of them have been approved for clinical trials [124, 125]. It is predicted that a multitude of vaccines based on bacterial EVs, with low toxicity and high efficiency, will be developed in future clinical trials [105]. Besides, control of the particle properties of EV-based vaccines may improve their immunization effects. During the immunization process, maturation of dendritic cells and presentation of antigens in lymph nodes are crucial for provoking a strong antigen-specific immune response. This immunization process can be modulated by the properties of vaccine particles, such as their size, rigidity, and shape [126]. The size of vaccine particles determines their trafficking mode from the site of administration to the lymph nodes. Vaccine particles with a size of 20-100 nm are mainly transferred to lymph nodes through lymphatic circulation, and larger particles are differently encapsulated and carried to lymph nodes by antigen-presenting cells [127]. Since the properties of synthetic nanoparticles are finely adjustable, nanoparticle-based bacterial EV vaccines can provoke a more robust antigen-specific immune response. Some examples of nanoparticle-based vaccines include coated bacterial EVs onto gold nanoparticles (BM-AuNPs), coated bacterial EVs onto bovine serum albumin nanoparticles (BN-EVs), and loading bacterial EVs into nanoparticles (NP-EVs), which are more stable and stronger immunostimulants than pure bacterial EVs [128–130]. Overall, different types of nanoparticles show different properties, and selection of the finest nanoparticles can augment the immunization of bacterial EV-based vaccines.

On the other hand, due to EV extraction from bacteria and their structural similarity, these components may act as decoys against bacteriophages. Bacteriophages may bind to LPS and be neutralized. This phenomenon decreases the potential efficacy of bacterial EVs and, subsequently, reduces their therapeutic efficacy. Some electron microscopy evidence confirms this finding for *E. coli*, *Salmonella*, and *V. cholera* [131, 132]. Some strategies have been suggested to overcome this limitation. The first strategy is concealing bacterial EVs with antifouling agents, such as poly(ethylene glycol), to reduce their immunological recognition by inhibiting protein binding [109]. The second strategy is to use complement system inhibitors to improve bacterial EV detection by the host cells. Such inhibitors are coated on the EV surface or administered before EV inoculation [133, 134]. The final approach is mimicking biological systems that may be potentially useful to evade recognition by immune cells. It seems that preparation of a hybrid membrane covered with bacterial EVs, along with some host cell membranes, such as platelets, leukocytes, and red blood cells is possible [135, 136].

Additionally, bacterial EVs mimic a bacterial structure to competitively bind to the target cells and suppress adhesion and infection caused by the main pathogens. Initiation of various infections is often induced by bacterial adhesion to target cells; therefore, antiadhesion therapies can decelerate the progression of infection [137, 138]. On the other hand, nonadhering infectious agents are recognized and neutralized more efficiently by immune cells [139]. Due to the presence of various intact bacterial adhesions on the EVs, these vesicles can be useful in antiadhesion therapies.

In the literature, the blockade of *H. pylori* adhesion to gastric epithelial cells has been reported by application of *H. pylori*-derived EVs [140]. Today, the potential applications of bacterial EVs, besides their known traditional applications, are being clarified. For example, use of these bioactive molecules in biosensing and biomedical imaging applications is becoming an interesting topic in biotechnology sciences [141]. Overall, the application of bacterial EVs is far more extensive and needs to be investigated in various contexts.

## 8. Conclusion

The current knowledge of bacterial EVs is very limited considering the vast spectrum of bacterial EVs in host-bacterial and interbacterial interactions, and further research is warranted. The investigation of bacterial EVs under different homeostatic and pathogenic conditions can also resolve many problems concerning host susceptibility. The importance of EVs derived from bacterial cells, associated with bacterial infection types and acute/chronic infections, has been also recently highlighted.

Overall, shedding of EVs is related to the adjustment of bacterial populations to unfavorable or changing conditions and controls the interaction of bacteria with their host cells and other bacteria. The ability of bacterial EVs to pass through epithelial, endothelial, and blood-brain barriers emphasizes the potential advantages of these delivery molecules in the targeted therapy of various infections, diseases, and disorders. Some bacterial EVs even engage in a mechanism of epigenetic modification, antibiotic resistance, and immune escape strategy. Moreover, the evaluation of EVs from commensal bacteria or different pathogens can provide an opportunity to improve personalized medicine in the near future.

## Figures and Tables

**Table 1:** The characteristics of different types of bacterial EVs.

Bacterial EV type	Derived from Gram-positive/Gram-negative bacteria	Derived from viable cells/cells lysis	Origination characteristics
OMV^1^	Gram-negative	Viable cells	Formed from outer membranes by budding/containing LPS, periplasmatic and cytosolic proteins, RNA and DNA, and virulence factors/A specialized bacterial secretion pathway
IMV^2^	Gram-negative	Viable cells	Formed by fission of a protrusion of the outer and plasma membranes
O-IMV^3^	Gram-negative	Viable cells/cells lysis	Formed as double bilayer EVs by cytoplasmic turgor pressure (frequently after cell lysis) which originally contain most DNA fragments and cytoplasmic contents
EOMV^4^	Gram-negative	Cells lysis	Formed by reassemble of membrane fragments after cell lysis and explodes/containing most DNA fragments and cytoplasmic contents
TSMS^5^	Gram-positive/Gram-negative	Viable cells	Formed from outer membranes in Gram-negative bacteria and unable to transfer cytoplasmic contents/formed from cytoplasmic membranes in Gram-positive bacteria and able to transfer cytoplasmic contents/an intercellular connection between neighboring cells to facilitate cellular components exchange
CMV^6^/microvesicle	Gram-positive	Viable cells/cells lysis	Formed by pressure, blebbing, or cell lysis from the cell wall
Bacterial EV derived by phage endolysin-triggered cell lysis	Gram-positive/Gram-negative	Cells lysis	Formed by enzymatic action that lyse the origin cells by phages
Bacterial EV derived from “hot spot” regions	Gram-positive/Gram-negative	Viable cells	Formed from specific regions that locally enriched with specific lipids and proteins involved in hypervesiculation
Bacterial EV derived under specific conditions	Gram-positive/Gram-negative	Viable cells/cells lysis	Formed by induced extended turgor pressure, membrane protuberances, and pinching-off of small membrane portions after accumulation of peptidoglycan or misfolded proteins in the periplasm/release of additional potential proteins into the extracellular space to combat stressors and survive

^1^Outer membrane vesicles, ^2^inner membrane vesicles, ^3^outer-inner membrane vesicles, ^4^explosive outer membrane vesicles, ^5^tube-shaped membranous structures, and ^6^cytoplasmic membrane vesicles.

## Data Availability

No data were used to support this study.
